# Mitigation of heat stress effects on laying hens' performances, egg quality, and some blood parameters by adding dietary zinc-enriched yeasts, parsley, and their combination

**DOI:** 10.3389/fvets.2023.1202058

**Published:** 2023-06-15

**Authors:** Gabriela Maria Cornescu, Tatiana Dumitra Panaite, Arabela Elena Untea, Iulia Varzaru, Mihaela Saracila, Mihaela Dumitru, Petru Alexandru Vlaicu, Teodor Gavris

**Affiliations:** ^1^Animal Nutrition Physiology Department, National Research and Development Institute for Biology and Animal Nutrition, Balotesti, Romania; ^2^Faculty of Animal Production Engineering and Management, University of Agronomic Sciences and Veterinary Medicine of Bucharest, Bucharest, Romania

**Keywords:** poultry, animal products, heat, parsley, yeast, minerals, eggs

## Abstract

**Objective:**

Finding natural, handy and efficient nutritional solutions to prevent and mitigate negative effects caused by environmental heat stress and to be applied to large-scale laying hen industry.

**Research design:**

A 3-weeks trial was conducted on 128 laying hens TETRA-SL LL (50 weeks of age) housed in 8 cages/group, 4 laying hens/cage, 32 laying hens/group, under heat stress conditions (34±1°C). The basal diet on corn and soybean meal was formulated to be isocaloric and isonitrogenic. Compared to Control group diet (C), experimental groups E1 included 1% zinc-enriched yeast; E2 included 2% parsley and E3 included 1% zinc-enriched yeast combined with 2% parsley to minimize the heat stress effects.

**Methods:**

The parsley and the zinc-enriched yeast were analysed for their chemical composition, total polyphenols, antioxidant capacity, minerals, vitamin E and incorporated into the ration structure. Production parameters, egg quality, biochemical and haematological profiles of blood samples were analysed during the trial.

**Results:**

A statistically significant (*p* < 0.05) average egg weight was noticed on E2 and E3 compared to Control group, and also during the 1st week compared to the 2nd and the 3rd experimental weeks. Average daily feed intake values were highly significant (*p* < 0.001) on E3 group compared to C, E1, E2, and on the 2nd week compared to the 3rd experimental week (*p* < 0.021). Feed conversion rate was highly significant (p < 0.001) during the 2nd and the 3rd experimental weeks compared to the 1st week. The average daily egg production was highly significant (*p* < 0.001) within 1st week compared to the 2nd and 3rd weeks. A highly significant (*p* < 0.001) yolk coloration was noticed on E2 and E3 groups. The malondialdehyde (MDA) concentration decreased significantly (*p* < 0.05) to all experimental groups compared to Control group during the 14th and 28th days of storage.

**Conclusion:**

These findings suggest that the two ingredients minimized the heat stress effects on production performance parameters with a demonstrated antioxidant capacity role by delaying the lipid peroxidation during different storage time periods.

## 1. Introduction

High temperature is one of the major environmental stressors in poultry production causing an increased vulnerability according to their breed, age, and genetic potential or nutritional status (1, 2). Heat stress decreases feed intake, egg production, and quality and increases the mortality rate of laying hens (3–6). Heat stress affects negatively intestinal barrier integrity and causes reduced nutrient absorption in laying hens which may be related with the imbalance of the gut microbiome (7).

The thermoneutral zone which allows optimal performances of laying hens is considered to be between 19°C and 22°C (8). At cellular level, heat stress increases reactive oxygen species which determines lipid peroxidation processes (9). Therefore, there is evidence that using plants with highly antioxidant potential and inhibitory activity of free radicals plays a crucial role in mitigating heat stress effects (10). Other several flock management strategies to overcome the deleterious effects of heat stress can be evaluated as tailored structural modifications–ramps and/or removing vertical barriers to increase freedom of movement (11), nutrient supplementation with different phytoadditives, vitamins and minerals (12), or breeding selection to promote heat tolerant poultry (13).

As some authors (14) stated is crucial to mitigate heat stress impact on poultry production and welfare by examining careful and controlling environmental conditions, taking into consideration that public poultry welfare concern and awareness increased.

Poultry, particularly during the final stages of their life cycle, exhibit high susceptibility to heat stress and pathogenic agents, leading to reduced feed intake and substantial impact on both their welfare and productivity. The perception of thermal discomfort may be noticed through the examination of animal behavioral disturbances, such as pecking or aggressiveness, search for cooler environments, restricted mobility, and wing spreading (15).

Parsley (*Petroselinum crispum*) contains essential mineral salts, iron, calcium, phosphorus, and vitamins A and C (16). In addition, some authors (17) confirmed that parsley is of great importance in preventing cell oxidation and developing the immune system, due to a high content of vitamin C. Parsley leaves contain a very important oil called myristicin with anti-inflammatory, analgesic, antiproliferative, and highly effective as antibiotics for negative bacteria and some fungi (18). Additionally, others authors (19, 20) considered parsley as an important source of redox-active compounds (ascorbic acid and carotenoids) and phenolic compounds with an antioxidant potential as flavones apigenin, luteolin, and gallic acid. Yeast, mostly brewer's yeast, has been appreciated as a high content of vitamin B complex; therefore, it was fed to the animals for many years now (21). Animals have been fed various forms of yeast and yeast derivatives for more than 100 years (22).

When the EU banned the use of antibiotics as growth promoters in animal feed on 1 January 2006, it was viewed as a difficulty at first and later as an opportunity to seek out novel feeding strategies and alternative products (23). Nowadays, beginning on 28 January 2022, a new European Law declared to be illegal using antibiotics to compensate for poor farm animal welfare standards.^1^ According to Azad et al., (24) *Saccharomyces* strains have the potential to accumulate high concentrations of copper (Cu), zinc (Zn), and manganese (Mn) salts to obtain ions enriched. Zn is often associated with oxidant defense system, part of Cu/Zn superoxide dismutase (SOD), a very important cellular defense against oxidative stress (25). Zn is regarded as a key component of redox metabolism in animal nutrition (26), ensuring the activation of over 200 distinct enzymes involved in protein metabolism and immunological function (27). Although it is believed that all Zn oxide sources were banned in EU since 2022 to reduce environmental impact, European Commission limits at 150 ppm total Zn utilization in complete feed; therefore, zinc oxide diet supplementation remains authorized (28). Rhodotorula glutinis biomass represents a good source of protein, lipids, and vitamins with positive effects on animal growth performance, their intestinal integrity, and immune system (29, 30). Production of yeast biomass rich in organically bound Zn is important to the animal industry because such forms of Zn are readily absorbed by the animal.

The current study aimed to evaluate the mitigating effects of dietary parsley and Zn-enriched yeast, individually and also combined on laying hens' productive performances, egg quality, and blood parameters when exposed to heat stress conditions.

## 2. Materials and methods

### 2.1. Parsley and zinc-enriched yeasts

The parsley (*Petroselinum crispum*) was purchased from a local Romanian phytopharmacy, already dried and over ground. To obtain zinc-enriched biomass, *Rhodotorula glutinis* CCY 020-002-033, yeast biomass has to be grown in culture medium supplemented with optimal concentration of zinc. This yeast strain was isolated from willow leaves, grown in synthetic medium at batch scale level, at optimal growth conditions, to assess its application as poultry feed additive. *Rhodotorula glutinis* CCY 020-002-033 yeast (RG) was provided by ICCF (Chemical and Pharmaceutical Research Institute of Bucharest). The strain was enriched by different percentages of zinc oxide (ZnO) (from 1 to 10%, w/v) on to Yeast extract Peptone Dextrose (YPD, g/L: 10 yeast extract, 20 peptone, 20 dextrose, 15–20 agar). RG was incubated at 28°C for 24–72 h to assess its application in poultry feed. The optimum ZnO selected inclusion rate was 10%, and our product registered a concentration of 3.25 x 107 col/g product with a level of 16.9 g Zn/100 g yeast.

### 2.2. Animals and experimental design

The experiment was conducted according with Directive 2010/63/EU, Executive Order no. 28/31.08.2011, Romanian law no. 43/11.04.2014. The experimental protocol no.118/02.12.2019 was approved by the Ethics Committee on Animal Experiments from the National Institute for Research and Development of Animal Biology and Nutrition, Balotesti, Romania. A total of 128 laying hens TETRA-SL LL (50 weeks of age) were weighed individually at the beginning of the experiment (1,660 ± 83.46 g) and assigned in a completely randomized design composed of four treatments, 8 cages/group, 4 laying hens/cage, and 32 laying hens/group for a 4-week trial (1-week accommodation period and 3 experimental weeks). The laying hens were previously raised as pullet in a local farm in Zucami improved cages. The cages are designed to provide poultry-friendly egg production, taking into consideration the health, comfort, and sustenance of the laying flocks, as well as the maintenance of high egg production rate. At 18 weeks age old, the poultry were purchased by Laboratory of Animal Physiology and Nutrition, INCDBNA—Balotesti, and transferred in adapted Zucami cages (provided with green plastic partitions which calm and reduce birds stress, with holes for optimum ventilation model F60 610, cage dimensions: front 610 mm; back 745 mm, height (mm: front 560/back 450, between levels 688, inclinations 8°/14%), suitable for digestibility trials, in order to experiment different phytoadditives in thermoneutral conditions or in extreme temperature conditions. To be ensured that the hens consumed only from designated feeder, a plastic divider was placed between the feeders. Water and feed access were provided *ad libitum*. The nipple drinkers were located so that each cage of birds had access on two drinkers. Twice per day water tank containers were checked to avoid water overheating and provide a water temperature of 18 ± 3°C. A week of adaptation period was assured prior to the beginning of the experiment (experimental diets were fed, and the hall temperature was risen gradually by 2°C every 2 days, 26°C, 28°C, 30°C, and 32°C, respectively to avoid heat–shock). When the experimental trial began, for heat stress evaluation, the hall temperature was set at 34 ± 1°C during the entire experimental period (24/24 h) by using a Big Dutchmann, ViperTouch computer system. The relative humidity of the experimental hall was registered between 40 and 45%. The lighting schedule used was the continuous lighting program of 16-h light and 8-h darkness per day. No antibiotics were provided during the entire trial experiment. All birds remained healthy during the whole trial.

### 2.3. Diet formulation

The basal diets were formulated according to the commercial requirements for TETRA-SL hybrid with a structured diet based on corn and soybean meal, and all diets formulated were isocaloric and isonitrogenic (Table 1). Compared to C diet, the other three experimental diets included different levels of Zn-enriched yeast and parsley, individually and combined, as follows: E1 included 1% Zn-enriched yeast; E2 included 2% parsley, and E3 group included 1% Zn-enriched yeast combined with 2% parsley. The diets were optimized using the nutritional feeding dedicated software Futter 2008, Hybrimin (GmbH & Co., Hessisch Oldendorf, Germany).

**Table 1:** Proximal chemical composition of the diets.

**Ingredients (%)**	**C**	**E1**	**E2**	**E3**
Yellow corn ground, %	44.68	43.51	43.09	41.93
Wheat, %	15.00	15.00	15.00	15.00
Soybean meal, 48 %	25.74	25.70	24.96	24.91
Vegetable oil, %	1.55	1.76	1.89	2.10
Methionine, %	0.15	0.15	0.17	0.17
Limestone, %	10.13	10.14	10.14	10.14
Phosphate, %	1.33	1.32	1.34	1.33
NaCl, %	0.37	0.37	0.37	0.37
Choline, %	0.05	0.05	0.05	0.05
Premix^*^, %	1.00	1.00	1.00	1.00
Yeast, %	-	1.00	-	1.00
Parsley, %	-	-	2.00	2.00
Total, %	100	100	100	100
**Calculated nutrient composition**				
Dry matter, %	88.78	88.79	89.06	89.07
Metabolizable energy, kcal/kg	2,750	2,750	2,750	2,750
Crude protein, %	17.10	17.10	17.10	17.10
Ether extract, %	2.24	2.20	2.20	2.16
Ash, %	2.52	2.52	2.45	2.45
Crude fiber, %	2.96	2.96	3.06	3.05
Digestible protein, %	15.14	15.14	14.73	14.72
Calcium, %	4.19	4.19	4.19	4.19
Available phosphorus %	0.38	0.38	0.38	0.38
Ca/P ratio	11.03	11.03	11.03	11.03
Sodium, %	0.16	0.16	0.16	0.16
Chloride, %	0.27	0.27	0.27	0.27
Lysine, %	0.90	0.90	0.87	0.87
Methionine, %	0.43	0.43	0.44	0.44
Methionine and cysteine, %	0.73	0.73	0.73	0.73
Threonine, %	0.66	0.66	0.64	0.64
Tryptophan, %	0.20	0.20	0.19	0.19
Arginine, %	1.06	1.06	1.03	1.03
**Analyzed nutrient composition**				
Zinc, mg/kg	128.89	390.17	106.53	379.29
Iron, mg/kg	426.57	372.29	405.40	384.60
Copper, mg/kg	19.82	12.71	14.12	14.21
Manganese, mg/kg	105.51	122.38	111.70	108.05
Lutein and zeaxanthin, ppm	7.14	9.18	11.02	8.40
Vitamin E, ppm	67.94	87.43	65.38	48.54
Total polyphenols, mg GAE/g	1.60	1.44	1.95	1.73
DPPH, mM equiv Trolox	8.02	7.82	8.46	8.97

C—conventional diet, E_1_–C+1% Zn-enriched yeast, E_2_–C+2% parsley, E_3_–C+1% Zn-enriched yeast and 2% parsley.

^*^Premix content per kg diet: vitamin A: 13.500 IU, vitamin D_3_: 3000 IU, vitamin E: 27 mg, vitamin K_3_: 2 mg, vitamin B1: 2 mg, vitamin B_2_: 4.8 mg, pantothenic acid: 14.85 mg, nicotinic acid: 27 mg, vitamin B_6_: 3 mg, vitamin B_7_: 0.04 mg, vitamin B_9_: 1 mg, vitamin B_12_: 0.018 mg, vitamin C: 25 mg, manganese: 71.9 mg, iron: 60 mg, copper: 6 mg, zinc: 60 mg, cobalt: 0.5 mg, iodine: 1.14 mg, selenium 0.18 mg; GAE, gallic acid equivalents; DPPH, 2,2-diphenyl-1-picrylhydrazyl; mM equiv Trolox, trolox equivalent antioxidant capacity.

### 2.4. Productive parameter evaluation

The birds were weighted at the beginning and at the end of the trial. During the experimental period, the productive parameters were evaluated, as follows: Laying rate (eggs number/hen/day) and average egg weight (AEW, g) were recorded daily, and average daily feed intake (ADFI, g/hen/d), feed conversion rate (FCR, g feed/g eggs), average daily egg production (ADEP, %), average egg weight (AEW, g), and average egg mass (g) were calculated daily. Feed conversion ratio (FCR) was calculated as the amount of feed consumed (g) required to produce a unit (g) of egg mass (g feed/g egg). Feed intake (g feed/bird/day) was reported by determining the difference between the total daily feed given to each cage with the feed refusals collected and weighted. Egg production (%) was collected daily at the same time 11.30 a.m., calculated on per cage basis over the entire experimental period and weighed every day. The egg classification was done according to Council Directive (2006) into four categories of eggs: extra-large (>73 g), large (73–63 g), medium (63–53 g), and small (< 53 g).

### 2.5. Blood sample collection

At the end of the trial (53 weeks of age), blood samples were collected from axillary vein (6 randomly chosen hens/group) into 6 ml lithium heparin coated vacutainer tubes to determine hematological and biochemical parameters analysis. The blood samples were centrifuged at 2,700 RPM for 20 min. After blood centrifugation, the serum was transferred in sterile tubs (Eppendorf, 2 ml) and kept at−20^0^C until the analysis. Plasma concentrations of glucose (GLU), cholesterol (CHOL), triglycerides (TG), total protein (TP), albumin (ALB), total bilirubin (TB), urea nitrogen (BUN), creatinine (CR), uric acid (UA), calcium (Ca), phosphorus (P), iron (Fe), and concentrations of alanine aminotransferase (ALT), aspartate aminotransferase (AST), alkaline phosphatase (ALP), and gamma glutamyl transferase (GGT) were analyzed using a spectrophotometer ABX PETRA 400 (HORIBA Medical, France). Hematological profile: hematocrit (HCT), leukocyte (LEUK), heterophyle (HET), lymphocyte (LYM), monocyte (MON), and eosinophil (EOS), was determined using ADVIA 2120i – Siemens (flow cytometry with peroxidase reaction and laser detection). Optical microscopy was used for the examination of blood smear.

### 2.6. Egg sampling and measurement procedures

A total number of 288 egg samples for the entire experimental period were collected randomly and weighed individually for determination of the external and internal egg quality. Eggs from each treatment were analyzed on first experimental week in stress conditions and on the final week of the experiment. Half of the collected eggs samples were analyzed for quality traits (whole egg, yolk, albumen and eggshell weight, pH albumen and yolk, albumen and yolk temperature, Haugh Unit, and yolk color) and antioxidant profile (minerals, vitamins, lutein, and zeaxanthin), while the other half of samples were collected and kept at room temperature for storage period analyses to assess the oxidative stability of yolk. For internal and external physical parameter measurements, each egg was individually weighed using the high-quality precision balance Kern EW6000-1M with a weighing capacity of max. 6,000 g/min. 5 g, readability 0.1 g (Kern & Sohn GmbH, D-72336 Balingen, Germany). After weighing, the eggs were broken and the egg components (albumen, yolk, and eggshell) were manually separated and weighed using the same balance as for the whole egg.

The assessment of pH albumen and yolk was measured using a portable pH meter (Five Go F2-Food kit with LE 427IP67, Sensor Mettler Toledo, Greifensee, Switzerland). We first proceed to standardize pH meter using buffer solution of 4.01 and 9.20. After being rinsed with deionized water, the electrode was dipped into the homogenate, sufficient time to stabilize before the reading the values. Haugh unit score was determined using) formula: HU= 100 x log (h-1.7w^0.37^+7.6), where HU—Haugh Unit, h—albumen height (mm), and w—egg weight (g) (31). Albumen height measurement was established using a digital caliper. To assess the storage period (0, 14, and 28 days), the egg weight loss (EWL) was calculated by multiplying average egg weight by egg week production and divided by 100 according to the Feddern's formula (32): Weight loss percentage (%) = [(initial egg weight - after storage egg weight)/(initial egg weight)] × 100.

### 2.7. Color parameter assessment

The yolk color was measured by a portable color spectrophotometer 3nh YS3020 (Shenzhen Threenh Technology Co., Ltd, Beijing, China) with customized aperture (8 mm/4 mm/1 × 3 mm), 2.6s measuring time, high accuracy of 0.04, with an observer angle of 2°/10° using the CIE-Lab system (Commission Internationale de l'Eclaraige). All measurements were performed in triplicate. The lightness L^*^ (with 0 as perfect black; 50 middle gray; 100 perfect white), the saturation index in green/red intensity a^*^ (negative values are green; positive values are red; 0 is neutral), and the saturation index in blue/yellow intensity b^*^ (negative values are blue; positive values are yellow; 0 is neutral) were determined by reflectance CIE—L^*^ a^*^ b^*^ color coordinates following the methods published by Panaite (33). In addition, the yolk color was measured by the Roche yolk color fan (Hoffman-La Roche Ltd., Basel, Switzerland; color scale from 15, dark orange, to 1, light pale).

### 2.8. Zinc and other mineral determination

Flame atomic absorption spectrometry [Thermo Electron—SOLAAR M6 Dual Zeeman Comfort (Cambridge, UK)] was used for mineral determination in raw materials, feeds, and eggs (3 eggs/samples, 6 samples/group) (34). The results were expressed as μg/g (ppm) of dried sample. The phosphorus (P) was determined spectrophotometrically according to Regulation (EC) no. 152/2009, using a molecular absorption spectrophotometer, Able Jasco V-530, Romania.

### 2.9. Content of total polyphenols

A modified Folin–Ciocalteu method (35) was used for spectrometrically determination of total polyphenols in vegetal materials. The reading of absorbance was performed at 732 nm, and the gallic acid was used for calibration curve, the results being expressed as mg gallic acid equivalents per gram sample.

### 2.10. Assay of 2,2-Diphenyl-1-picrylhydrazyl for measuring antioxidant capacity

The antioxidant activity of vegetal materials was measured by 2,2-Diphenyl-1-picrylhydrazyl (DPPH) radical-scavenging activity according to the method described by Untea (36). The absorbance was read at 517 nm using a spectrophotometer (Jasco V-530, Japan Servo Co. Ltd., Japan). The results were expressed as mM eq trolox after a standard calibration curve was constructed by plotting percentage inhibition against trolox different concentrations.

### 2.11. Yolk oxidative stability by thiobarbituric acid reactive substances

The oxidative stability of yolk was indicated by the levels of thiobarbituric acid reactive substances (TBARSs), according to the methods described by Untea (35). The TBARS values were calculated from a standard curve of malondialdehyde and expressed as milligrams of malondialdehyde (MDA) per kg of sample (mg MDA/kg). The absorbance of the prepared sample was read at 532 nm.

### 2.12. Lutein and zeaxanthin determination

For lutein (LUT) and zeaxanthin (ZX) determination, an RP HPLC analytical method was used as described by Vărzaru (37). A high-performance liquid chromatograph (Perkin Elmer 200 series, Shelton, CT, USA) with a UV detector (445 nm) and a stationary phase of 5 μm C18 reversed-phase column of 250 × 4.60 mm (Nucleodur, Macherey-Nagel, Germany) was used. Chromatographic parameters were as follows: flow rate of 1.0 mL/min and a mobile phase of 13% water and 87% acetone.

### 2.13. Vitamin E determination

For vitamin E, determination was used an RP HPLC analytical method described by Vărzaru (37). A high-performance liquid chromatograph (HPLC Finnigan Surveyor Plus, Thermo-Electron Corporation, Waltham, MA), a PDA-UV (292 nm) with a Hypersil BDS C18 column, with silica gel, dimensions of 250 × 4.6 mm, and a particle size of 5 μm (Thermo-Electron Corporation, Waltham, MA), was used. Chromatographic parameters were as follows: flow rate of 1.5 mL/min and a mobile phase of 4% water, using 96% methanol.

### 2.14. Statistical analyses

Each cage of five birds was considered as an experimental unit. The normal distribution of data was checked by Kolmogorov–Smirnov and Shapiro–Wilk tests. Differences between groups were analyzed with one-way analysis of variance (ANOVA) by using the statistical program IBM SPSS 20.0. Tukey's comparison test was used to evaluate statistical significance of differences between dietary treatments. The significant differences among treatments were determined at probability of *p* < 0.05.

## 3. Results

### 3.1. Zinc-enriched yeast and parsley chemical characterization

The parsley and zinc-enriched yeast samples were analyzed to determine their proximal chemical composition, total polyphenols content, and their antioxidant capacity as experimental dietary ingredients, and the results are presented in Table 2.

**Table 2:** Proximal chemical composition and antioxidant profile of parsley and zinc-enriched yeasts.

**Specification**	**Parsley**	**Zn-enriched yeast**
**Proximal chemical composition**		
Dry matter, %	90.06	91.63
Organic matter, %	69.89	74.84
Crude protein, %	23.49	10.82
Ether extract, %	1.40	0.54
Crude fiber, %	8.73	2.21
Ash, %	20.17	16.79
**Total polyphenols, antioxidant capacity, minerals, and vitamin E**		
Total polyphenols, mg GAE/g	7.71	0.40
DPPH, mM equiv. Trolox	13.05	1.25
Zinc, mg/kg	56.16	97,105.59
Iron, mg/kg	3,817.70	1,619.25
Copper, mg/kg	12.64	302.84
Manganese, mg/kg	121.59	454.53
Vitamin E, mg/kg	25.33	3.61

GAE, gallic acid equivalents; DPPH, 2,2-diphenyl-1-picrylhydrazyl; mM equiv. Trolox, trolox equivalent.

The parsley's chemical characterization presented a higher content of crude protein, crude fiber, total polyphenols, antioxidant capacity, and iron concentration compared to Zn-enriched yeast composition. On the contrary, the Zn-enriched yeast composition registered a higher content of trace minerals (zinc, copper, and manganese) compared to parsley. Both ingredients present antioxidant potential due to their content in minerals, polyphenols, and vitamin E which protects against oxidative damage.

### 3.2. Productive performances

The effects of dietary parsley, Zn-enriched yeast, and the two mixed plant ingredients on laying hens performances are presented in Table 3.

**Table 3:** Effect of diet supplementation by Zn-enriched yeast (1%), parsley (2%), and mixed plant additive ingredients on productive performances under heat stress conditions (weeks 50–53 of age).

**Period trial/group**	**ADFI (g/day/hen)**	**FCR (g feed/g eggs)**	**ADEP (%)**	**AEM (g)**	**AEW (g)**	**Egg size classification** ^*^		
						**S %**	**M %**	**L %**
**Week 1 (50–51 wks)**								
C	63.43^b^	1.57	76.43	44.00	57.54^b^	17.42^a^	69.43	13.15
E1	65.46^ab^	1.62	72.14	42.10	58.39^ab^	8.33^b^	72.24	19.43
E2	68.95^ab^	1.80	72.93	42.67	58.52^ab^	19.79^a^	60.45	19.56
E3	71.28^a^	1.65	77.44	45.71	58.90^a^	13.27^ab^	68.93	17.80
SEM	1.27	0.04	1.79	1.60	0.22	1.31	2.10	1.44
**Week 2 (51–52 wks)**								
C	61.93^b^	1.96	62.14	34.76	55.76	25.17	62.57	12.26
E1	62.07^b^	1.76	67.14	38.04	56.61	21.26	65.42	13.32
E2	66.28^b^	2.06	66.92	38.18	57.04	18.27	68.05	13.68
E3	73.53^a^	1.97	69.92	39.82	56.83	22.28	62.98	14.74
SEM	1.17	0.08	2.00	1.21	0.27	2.20	2.41	1.31
**Week 3 (52–53 wks)**								
C	66.86^ab^	2.26	60.71	33.80	55.60^b^	25.12	67.06	7.82^b^
E1	67.43^b^	1.95	70.00	38.91	55.64^b^	27.83	62.30	9.87^ab^
E2	69.14^ab^	2.21	62.41	35.22	56.63^ab^	23.89	58.60	17.51^a^
E3	74.40^a^	2.04	66.92	38.29	57.26^a^	23.27	59.59	17.14^a^
SEM	1.08	0.11	2.59	1.43	0.28	2.13	2.65	1.69
**Overall (50–53 weeks)**								
C	64.74	1.93^ab^	66.43	37.52	55.60	25.12	67.06	7.82
E1	64.99	1.78^b^	69.76	39.68	55.64	27.83	62.30	9.87
E2	68.12	2.03^a^	67.42	38.69	56.63	23.89	58.60	17.51
E3	73.07	1.89^ab^	71.43	41.27	57.26	23.27	59.59	17.14
SEM	6.40	0.05	12.05	7.26	0.14	1.01	1.89	2.49
**Main effects**								
**Group**								
C	64.74^C^	1.93	66.43	37.52	56.30^b^	22.57	68.31	11.08^b^
E1	64.99^CB^	1.78	69.76	39.68	56.88^ab^	19.14	70.49	14.21^ab^
E2	68.12^B^	2.02	67.42	38.69	57.40^a^	20.65	68.45	16.92^a^
E3	73.07^A^	1.89	71.43	41.27	57.66^a^	19.61	64.94	16.56^a^
**Period**								
Week 1	67.28 ^ab^	1.66^B^	74.74^a^	43.62^A^	58.34^A^	14.71^b^	71.17	17.49^a^
Week 2	65.95^b^	1.94^A^	66.53^b^	37.70^B^	56.56^B^	21.75^a^	67.86	13.50^ab^
Week 3	69.96^a^	2.12^A^	65.01^b^	36.56^B^	56.28^B^	25.03^a^	65.10	13.09^b^
***p*** **-Value**								
Group	< 0.001	0.340	0.502	0.333	0.007	0.717	0.579	0.061
Period	0.021	0.001	0.005	0.000	< 0.001	0.003	0.222	0.064
Group^*^Period	0.613	0.948	0.800	0.865	0.857	0.564	0.732	0.653

C—conventional diet; E_1_–C+1% yeast; E_2_–C+2% parsley; E_3_–C+1% yeast and 2% parsley; data are presented as mean ± SEM; means within a column sharing different lower superscripts are significantly different at p < 0.05 or upper superscripts for statistically highly significant at p < 0.001; minimum mass per egg in European Union, small size (S) < 53g, medium size (M), 53–63g, large size (L) 63–73g, extra-large (XL)>73 g.

The ADFI values were statistically highly significant (*p* < 0.001) on group E_3_ compared to C, E_1_, and E_2_ groups. In addition, a statistically significant value (*p* = 0.021) of this parameter could be noticed within the 3rd experimental week compared to the 2nd experimental week. There were no statistically significant differences (*p* < 0.05) observed on FCR between groups, but statistically highly significant value (*p* < 0.001) was registered on the 3rd and the 2nd experimental weeks compared to 1st week. The ADEP was statistically highly significant (*p* < 0.005) within 1st week compared to the 2nd and 3rd weeks, but no statistically significant differences (*p* > 0.05) were observed between groups. As expected, the heat stress affected negatively the egg weight on C control group. Under heat stress conditions, the average egg weight was statistically significant (*p* < 0.05) for E_2_ and E_3_ groups compared to C, and also a statistically significant weight (*p* < 0.05) could be noticed on the 1st week compared with the 2nd and the 3rd week. The commercial size egg classification registered statistically significant values (*p* < 0.05) for L size eggs on E_2_ and E_3_ groups compared to C group. The percentage of L size eggs was statistically significant (*p* < 0.05) during the 1st experimental week whereas for S size egg classification registered statistically significant (*p* < 0.05) on the 2nd and the 3rd weeks.

### 3.3. Egg quality parameters during experimental period

As presented in Table 4 there were no statistically differences (*p* > 0.05) concerning the whole egg weight at the beginning of the experiment, although during the end of the experiment, the E_1_ and E_2_ eggs' weight was significant different (*p* < 0.05) compared to C and E_3_ groups. In addition, E_1_ and E_2_ registered significant differences (*p* < 0.05) on albumen weight and yolk weight compared to the other two groups. The pH albumen values registered significant differences (*p* < 0.05) at the beginning of the experiment on E_2_ and E_3_ groups compared to C and E_1_. The yolk color intensity values were statistically highly significant (*p* < 0.0001) both at the beginning and at the end of the experiment on E_2_ and E_3_ groups compared to C and E_1_. The results obtained analyzing trace mineral content data and antioxidant profile of egg yolk samples are presented in Table 5.

**Table 4:** Internal and external egg quality parameters at the beginning and at the final of the experiment.

**Group**	**Initial (50 weeks)**							
	**Whole egg weight (g)**	**Albumen weight (g)**	**Yolk weight (g)**	**Eggshell weight (g)**	**pH albumen**	**pH yolk**	**Yolk color**	**HU**
C	60.60	37.40	15.15^a^	7.65	9.00^b^	6.58	4.50^C^	97.52^a^
E1	60.91	38.10	15.35^a^	7.47	9.16^b^	6.58	4.28^C^	97.96^a^
E2	60.14	38.25	14.56^b^	7.33^a^	9.25^a^	6.59	6.06^B^	97.63^a^
E3	59.83	37.03	15.29^a^	7.50^b^	9.29^a^	6.50	6.67^A^	93.04^b^
SEM	0.252	0.221	0.106	0.055	0.025	0.013	0.104	0.599
*p*-value	0.749	0.534	0.121	0.220	0.004	0.311	< 0.0001	0.009
**Final (53 weeks)**								
C	57.17^b^	36.41	13.92	6.85	8.91	6.60	4.28^C^	95.02
E1	59.09^a^	37.61	14.12	7.36	9.01	6.58	4.29^C^	98.00
E2	59.62^a^	37.91	14.53^a^	7.18	9.03	6.63	6.44^B^	97.91
E3	55.83^c^	35.58	13.33^b^	6.91	9.00	6.59	7.11^A^	94.94
SEM	0.379	0.314	0.114	0.064	0.024	0.013	0.120	0.582
*p*-value	0.043	0.251	0.077	0.207	0.410	0.789	< 0.0001	0.120

C—conventional diet; E_1_–C+1% yeast; E_2_–C+2% parsley; E_3_–C+1% yeast and 2% parsley; data are presented as mean ± SEM; means were calculated using six replicates per treatment (three eggs per replicate); means within a column sharing different lower superscripts are significantly different at p < 0.05 or upper superscripts for statistically highly significant at p < 0.001.

**Table 5:** Antioxidant profile and trace minerals analysis of egg yolk samples at the final of the experiment.

**Specification**	**Vitamin E (mg/kg)**	**Vitamin A (mg/kg)**	**LUT&ZX (mg/kg)**	**TP, mgGAE/g**	**DPPH, mM Trolox**	**Fe (mg/kg)**	**Zn (mg/kg)**
C	46.70	5.913	4.313	0.706	1.917	171.3	75.35
E1	43.79	7.643	4.689	0.743	1.878	171.2	77.18
E2	55.59	7.013	8.336	0.765	2.553	163.9	70.44
E3	52.66	6.270	6.690	0.860	1.801	170.8	77.91
SEM	0.79	0.091	0.134	0.029	0.063	0.436	0.266
**Main effect**							
**Zn-enriched yeast**							
Without Zn-enriched yeast	51.15	6.46^b^	6.325^a^	0.736	2.255^a^	167.6^B^	72.89^B^
With Zn-enriched yeast	48.22	6.96^a^	5.690^b^	0.812	1.878^b^	171.0^A^	77.55^A^
**Parsley**							
Without parsley	45.24^B^	6.78	4.50^B^	0.735	1.930	171.3^A^	76.27^A^
With parsley	54.13^A^	6.64	7.5^A^	0.813	2.180	167.3^B^	74.18^B^
***p*** **-value**							
Zn-enriched yeast	0.079	0.014	0.028	0.205	0.009	0.001	0.000
Parsley	0.000	0.463	0.000	0.200	0.061	0.000	0.001
Zn-enriched yeast^*^parsley	0.993	0.000	0.001	0.751	0.006	0.001	0.000

C—conventional diet, E_1_–C+1% yeast, E_2_–C+2% parsley, E_3_–C+1% yeast and 2% parsley; data are presented as mean ± SEM; means within a column sharing different lower superscripts are significantly different at p < 0.05 or upper superscripts for statistically highly significant at p < 0.001; means were calculated using six replicates per treatment (three eggs per replicate); TP, total polyphenols; GAE, gallic acid equivalents; DPPH, 2,2-Diphenyl-1-picrylhydrazyl, trolox equivalent antioxidant capacity; Fe (iron); Zn (zinc).

The yolk sample analysis for vitamin E and LUT and ZX concentration confirmed statistically highly significant differences (*p* < 0.001) on E_2_ and E_3_ groups compared to E1 and C. Moreover, for DPPH values, statistically significant differences (*p* < 0.05) were noticed on E_3_ group compared to E_2_, E_3_, and C groups. Concerning iron concentration, statistically significant differences (*p* < 0.05) were registered on E_1_ and E_3_ groups compared to C and E_2_. Vitamin A concentration registered statistically highly significant differences (*p* < 0.001) within E1 and E3 groups compared to C and E_2_.

Table 6 presents internal and external egg quality data parameters during storage period (0, 14, and 28 days) at room temperature (21°C).

**Table 6:** Internal and external egg parameters during different storage time periods (0, 14, and 28 days) at room temperature.

**Specification**		**Egg weight and its components**				**Internal egg parameters**		
		**Whole egg (g)**	**Albumen (g)**	**Yolk (g)**	**Eggshell (g)**	**pH-yolk**	**pH-albumen**	**Haugh unit**
**0 days**								
C		57.17^ab^	36.41	13.92^ab^	6.85^b^	6.60	8.91	95.02
E1		59.18^a^	37.70	14.11^ab^	7.37^a^	6.58	9.01	98.11
E2		59.62^a^	37.91	14.53^a^	7.18^ab^	6.63	9.02	97.91
E3		55.82^b^	35.58	13.33^b^	6.91^b^	6.59	9.00	94.94
SEM		0.51	0.43	0.16	0.06	0.02	0.03	0.68
**14 days**								
C		56.92	34.83	14.86	7.23	6.71	9.66	83.06^b^
E1		56.75	34.68	14.81	7.26	6.57	9.54	82.41^b^
E2		56.04	34.24	14.95	6.84	6.64	9.57	89.64^a^
E3		56.58	33.65	15.56	7.37	6.75	9.34	85.58^ab^
SEM		0.59	0.50	0.21	0.10	0.05	0.06	0.83
**28 days**								
C		56.64^ab^	34.97^a^	14.42	7.25	6.59	9.52	73.61^b^
E1		55.21^ab^	33.48^ab^	14.76	6.97	7.06	9.37	76.05^ab^
E2		57.15^a^	34.27^ab^	16.03	6.85	6.94	9.58	78.42^ab^
E3		53.62^b^	31.93^b^	14.51	7.08	6.70	9.50	80.11^a^
SEM		0.58	0.46	0.42	0.12	0.08	0.05	0.90
**Main effect**								
Group	C	56.91^ab^	35.40^a^	14.40	7.11	6.63	9.36	83.90^c^
	E1	57.05^ab^	35.29^a^	14.56	7.20	6.74	9.31	85.53^b, c^
	E2	57.61^a^	35.47^a^	15.17	6.96	6.74	9.39	88.66^a^
	E3	55.31^b^	33.72^b^	14.47	7.12	6.68	9.28	86.88^a, b^
Period	0 day	57.95^a^	36.91^A^	13.97^b^	7.08	6.60^b^	8.98^B^	96.50^A^
	14 days	56.57^ab^	34.35^B^	15.05^a^	7.18	6.67^b^	9.53^B^	85.17^B^
	28 days	55.63^b^	33.66^B^	14.93^a^	7.04	6.82^a^	9.49^A^	77.05^C^
SEM		0.32	0.27	0.16	0.05	0.03	0.03	0.47
***p*** **-value**								
Group		0.077	0.068	0.285	0.475	0.587	0.434	0.004
Period		0.019	< 0.001	0.012	0.579	0.018	< 0.001	< 0.001
Group^*^period		0.309	0.698	0.466	0.172	0.112	0.336	0.095

C—conventional diet; E_1_–C+1% yeast; E_2_–C+2% parsley; E_3_–C+1% yeast and 2% parsley; data are presented as mean ± SEM; means within a column sharing different lower superscripts are significantly different at p < 0.05 or upper superscripts for statistically highly significant at p < 0.001; means were calculated using six replicates per treatment (three eggs per replicate).

**Figure 1:**
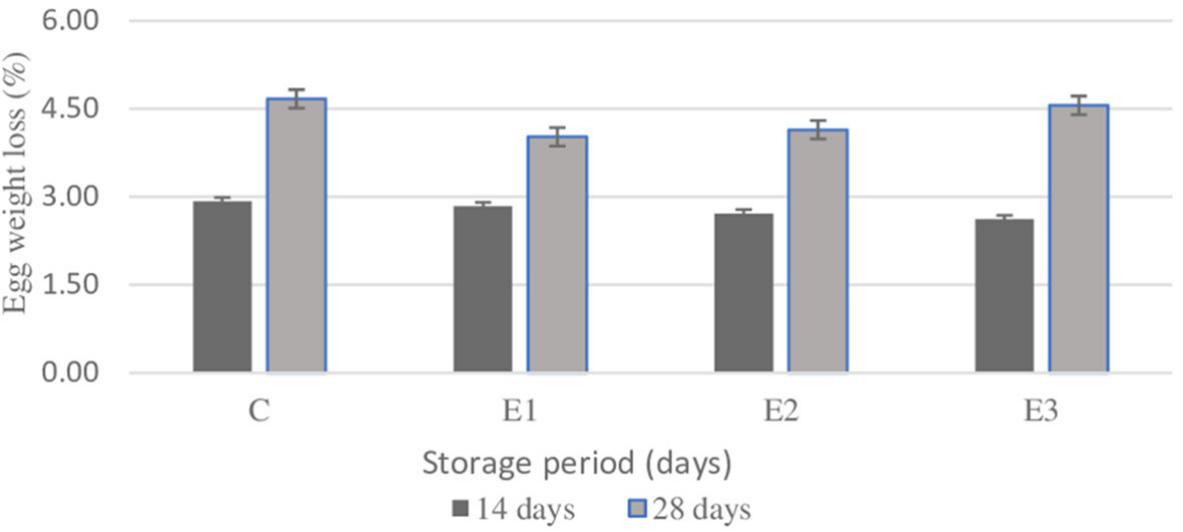
Effect of different lengths storage period of time (14 and 28 days) on egg weight loss (%). C, conventional diet; E1, C+1% yeast; E2, C+2% parsley; E3, C+1% yeast and 2% parsley; Data are presented as mean ± SEM.

The whole egg weight values were statistically significant (*p* < 0.05) for E_2_ group compared to E_3_ group. As expected, the egg weight values were statistically significant (*p* < 0.05) on 0 days of storage period compared to the whole egg weight registered at 28th days of storage. The egg albumen weight registered statistically significant values (*p* < 0.05) at C, E_1_, and E_2_ groups compared to E_3_ group. The same parameter registered the statistically highly significant value (*p* < 0.001) on 0 days of storage. The yolk weight values were statistically highly significant (*p* < 0.012) during the 28th days of storage compared to other 2 periods. There were no differences noticed between groups. The yolk pH values registered statistically highly significant value (*p* < 0.018) on the 28th storage period compared with the other two storage periods. The albumen pH values were statistically highly significant (*p* < 0.001) on the 14th and 28th storage period, without any statistical differences observed between groups. The HU score ranged between 83 and 88 between groups and 77 and 96 HU between periods. The Haugh unit was statistically highly significant (*p* < 0.004) at E_2_ compared to E_1_ and C groups. In addition, as expected, the Haugh unit exhibited a gradually decreasing curve consecutively with the increasing storage period. A statistically highly significant (*p* < 0.001) difference could be noticed during the 0 days of storage compared to the other two periods. The effects of different storage period of time on egg loss parameters values at room temperature (21±1°C) are shown in Figure 1.

There were no significant differences (*p* > 0.05) between groups at 14-day storage period, where an average of 2.77 % of weight loss was registered. Same situation was noticed on the 28-day storage period, where no statistical differences (*p* > 0.05) between groups were noticed. As expected, along with the length of storage also egg weight losses increase at 4.45 % at C and E_3_ groups. Within Table 7 are presented the color parameter values indicating yolk pigmentation evolution at room temperature during 14- and 28-day storage period.

**Table 7:** Yolk color evolution during different storage time periods at room temperature (21 ± 1°C).

**Parameter**		**Fan color**	L^*^	a^*^	b^*^
**0 days**					
C		4.28^d^	42.34	0.26^b^	14.84^b^
E1		4.31^cd^	42.15	0.26^b^	13.98^b^
E2		6.44^b^	41.04	0.96^a^	16.23^a^
E3		7.11^a^	41.12	0.71^a^	16.00^a^
SEM		0.07	0.36	0.06	0.22
**14 days**					
C		4.83^b^	45.62^a^	0.47^b^	17.39^b^
E1		5.83^a^	43.78^b^	0.38^b^	15.82^c^
E2		5.17^b^	43.30^b^	1.23^a^	18.81^a^
E3		5.33^ab^	43.27^b^	0.90^a^	18.08^ab^
SEM		0.11	0.30	0.07	0.24
**28 days**					
C		4.33^b^	50.24^b^	−0.01^b^	16.32^c^
E1		6.25^ab^	54.54^a^	1.24^a^	24.67^a^
E2		6.50^ab^	56.36^b^	1.30^a^	22.01^b^
E3		6.67^a^	54.60^b^	1.11^a^	24.86^a^
SEM		0.17	0.53	0.08	0.36
**Main Effect**					
Group	C	4.48^B^	47.62^a, c^	0.24^D^	16.18^D^
	E1	5.46^B^	45.39 ^b^	0.63^C^	18.16^C, B^
	E2	6.04^A^	46.29 ^b, c^	1.16^A^	19.01^B^
	E3	6.37^A^	46.92^c^	0.91^B^	19.65^A, B^
Period	0 days	5.54^b^	41.66^C^	0.55^B^	15.26^C^
	14 days	5.29^b^	43.99^B^	0.74^A, B^	17.53^B^
	28 days	5.94^a^	54.01^A^	0.91^A^	21.96^B^
SEM		0.07	0.24	0.04	0.16
***p*** **-value**					
Group		< 0.001	0.008	< 0.001	< 0.001
Period		0.002	< 0.001	0.002	< 0.001
Group^*^period		< 0.001	< 0.001	< 0.001	< 0.001

C—conventional diet (C); E1—C+1% yeast; E2—C+2% parsley; E3—C+1% yeast and 2% parsley; data are presented as mean ± SEM; means within a column sharing different lower superscripts are significantly different at p < 0.05 or upper superscripts for statistically highly significant at p < 0.001; means were calculated using six replicates per treatment (three eggs per replicate); L^*^ lightness, a^*^ redness intensity, b^*^ yellowness intensity.

An intense yolk coloration, statistically highly significant (*p* < 0.001) measured by Fan color, was noticed on groups within parsley inclusion E_2_ and E_3_, and statistically highly significant (p = 0.002) coloration intensity was registered within the 28th storage period. The yolk color saturation expressed by lightness (L^*^) parameter registered statistically highly significant (*p* = 0.008) at C group compared to E_1_ and E_2_ groups. As storage time concerns, statistically highly significant (*p* < 0.001) for the same parameter was registered on the 28th storage period compared to the other 2 periods. Concerning a^*^(redness intensity parameter), E_2_ group registered statistically highly significant (*p* < 0.001) compared to all other experimental groups, and as the storage time increased, the highest statistical value (*p* = 0.002) was noticed on 28th and 14th storage period. Values of b^*^ parameter (yellowness intensity) were statistically highly significant (*p* < 0.001) at E_3_ group compared to C, E_1_, and E_2_ while statistically highly significant differences (*p* < 0.001) were recorded on the 28-day storage period.

**Figure 2:**
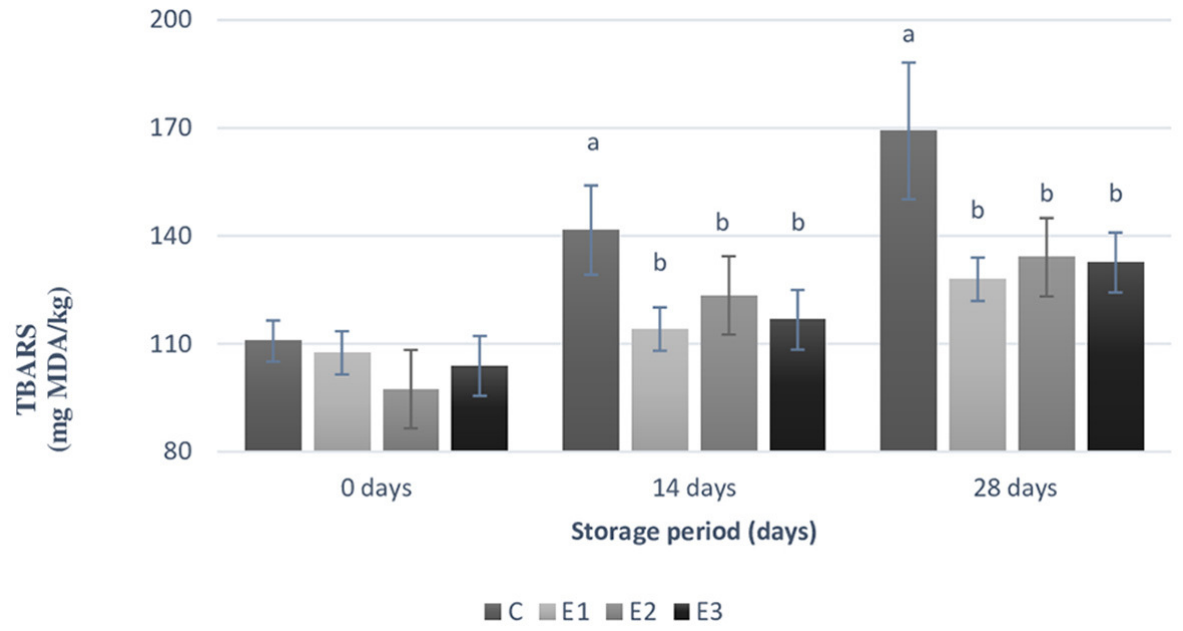
Levels of thiobarbituric acid reactive substances (TBARS) within yolk samples during different storage period (0, 14, 28 days) at room temperature (21°C). C, conventional diet; E1, C+1% yeast; E2, C+2% parsley; E3, C+1% yeast and 2% parsley; Data are presented as mean ± SEM. TBARS, thiobarbituric acid reactive substances expressed as milligrams of malondialdehyde (MDA) per kg of sample (mg MDA/kg); Means were calculated using 6 replicates per treatment, a, b Means with different superscripts differ significantly (*p* < 0.05).

According to Figure 2, the parley and yeast inclusion resulted in a statistically significant decrease (*p* < 0.05) in the MDA concentration to all experimental groups compared to C group, during both the 12th and 28th days of storage. An increasing of 53% of TBARS values was noticed at C group between 0 days of storage period and 28th days of storage, compared to 19% increasing of TBARS values at E1 group, 37% increasing of TBARS values at E_2_ group, and 28% increasing of TBARS values at E_3_ group. On the 14th day of storage, statistically significant difference (*p* < 0.04) was noticed between C and E_1_ groups. Same statistically significant difference (*p* < 0.04) between the same two groups was maintained during the 28th storage period.

### 3.4. Biochemical and hematological profile

Data on the serum biochemical indices are presented in Table 8. Dietary supplementation with Zn-enriched yeast, parsley, and their combination had no effect (*p* > 0.05) on serum concentration levels of GLU, CHOL, TG, ALB, CR, UA, BUN, TP, TB, ALAT, ALP, and ASAT during the trial period within our study, although a statistically significant difference (*p* < 0.05) was noticed on concentration level of minerals as phosphorus, on E_2_ group compared to C, E_1_, and E_3_ groups. Moreover, a statistically significant difference (*p* < 0.05) of Ca and Fe concentration levels was registered at E_2_ and C groups compared to E_1_ and E_3_. The GGT values were statistically significant (*p* < 0.05) on E_1_ and E_3_ groups compared to E_2_ and C groups.

**Table 8:** Diet supplementation effects with Zn-enriched yeast (1%), parsley (2%), and their combination on serum biochemical profiles under heat stress conditions (weeks 50–53 of age).

**Specifica-tion**	**Energy-related metabolites**			**Protein-related metabolites**						**Mineral-related metabolites**			**Enzyme-related metabolites**			
	**GLU (mg/dL)**	**CHOL (mg/dL)**	**TG (mg/dL)**	**TP (g/dL)**	**ALB (g/L)**	**TB (mg/dL)**	**BUN (mg/dL)**	**CR (mg/dL)**	**UA (mg/dL)**	**P (mg/dL)**	**Ca (mg/dL)**	**Fe (ug/dL)**	**ALT (U/L)**	**ALP (U/L)**	**GGT (U/L)**	**AST (U/L)**
C	178.24	86.02	701.13	1.89	1.40	0.13	0.67	0.32	3.38	4.14	23.85	209.38	9.91	719.60	82.24	87.20
E1	186.54	102.02	979.46	1.94	1.20	0.14	0.41	0.12	4.33	4.07	24.77	212.77	10.32	685.03	74.72	97.24
E2	177.45	87.86	712.52	2.00	1.25	0.13	0.47	0.12	3.17	5.39	21.47	200.56	10.32	674.17	99.90	91.00
E3	172.62	84.66	572.35	1.96	1.50	0.12	0.46	0.12	3.75	3.13	20.78	171.49	8.87	879.29	77.15	84.52
SEM	2.65	4.95	75.56	0.04	0.11	0.00	0.05	0.05	0.33	0.27	0.69	3.81	0.26	35.11	2.89	1.86
**Main effect**																
**Zn-enriched yeast**																
Without Zn-enriched yeast	177.84	86.94	706.82	1.94	1.33	0.13	0.57	1.94	3.28	3.60^a^	22.66	204.97	10.12	696.89	91.07^a^	89.10
With Zn-enriched yeast	179.58	93.34	775.91	1.95	1.35	0.13	0.44	1.95	4.04	4.76^b^	22.78	192.13	9.60	782.16	75.94^b^	90.88
**Parsley**																
Without Parsley	182.39	94.02	840.29	1.91	1.30	0.13	0.54	1.91	3.86	4.10	24.31^a^	211.08^a^	10.12	702.32	78.48	92.22
With Parsley	175.03	86.26	642.44	1.98	1.38	0.12	0.47	1.98	3.46	4.26	21.13^b^	186.02^b^	9.60	776.73	88.52	87.76
***p*** **-value**																
Zn-enriched yeast	0.75	0.53	0.65	0.96	0.91	0.66	0.17	0.96	0.26	0.04	0.94	0.11	0.33	0.24	0.02	0.64
Parsley	0.18	0.44	0.21	0.41	0.73	0.19	0.43	0.41	0.56	0.78	0.04	0.01	0.33	0.31	0.10	0.25
Zn-enriched yeast^*^parsley	0.23	0.35	0.19	0.58	0.31	0.40	0.20	0.58	0.79	0.06	0.57	0.05	0.09	0.11	0.21	0.04

C—conventional diet; E1—C+1% yeast; E2—C+2% parsley; E3—C+1% yeast and 2% parsley; data are presented as mean ± SEM; a-b means with different superscripts differ significantly (p < 0.05); glucose (GLU), cholesterol (CHOL), triglyceride (TG), total protein (TP), albumin (ALB), total bilirubin (TB), urea nitrogen (BUN), creatinine (CR), uric acid (UA), phosphorus (P), calcium (Ca), iron (Fe), alanine aminotransferase (ALT), alkaline phosphatase (ALP), gamma glutamyl transferase (GGT), and aspartate aminotransferase (AST).

As shown in Table 9 hematological analysis of blood samples had no apparent influence on selected hematologic values: LEU, LYM, MON, and EOS. Our results showed a statistically significant higher values (*p* < 0.05) of HCT for E_1_ and E_3_ groups compared to C and E_2_. In addition, a statistically significant higher values (*p* < 0.05) of HET were noticed on C and E_1_ groups compared to E_2_ and E_3_. Blood urea parameter significant increased (*p* < 0.05) on E1 group compared to C and E_2_ groups.

**Table 9:** Effect of diet supplementation by Zn-enriched yeast (1%), parsley (2%) and their combination on hematological values under heat stress conditions (weeks 50–53 of age).

**Specification**	**HCT (%)**	**LEUK (%)**	**HET (K/μL)**	**LYM (K/μL)**	**MON (K/μL)**	**EOS (K/μL)**
C	21.17	12.47	5.80	6.37	0.36	0.15
E1	24.67	12.67	5.71	6.58	0.24	0.15
E2	23.33	12.33	5.35	6.87	0.22	0.12
E3	25.40	15.12	6.60	7.77	0.34	0.53
SEM	0.501	0.388	0.145	0.217	0.051	0.017
**Main effect**						
**Zn-enriched yeast**						
Without Zn-enriched yeast	22.25^b^	12.40	5.57	6.62	0.290	0.137
With Zn-enriched yeast	25.03^a^	13.89	6.12	7.17	0.290	0.336
**Parsley**						
Without parsley	22.92	12.57	5.75	6.47	0.300	0.239
With parsley	24.37	13.73	5.92	7.32	0.280	0.137
***p*** **-Value**						
Zn-enriched yeast	0.012	0.069	0.387	0.215	1.000	0.000
Parsley	0.164	0.151	0.409	0.064	0.846	0.000
Zn-enriched yeast^*^ parsley	0.483	0.111	0.638	0.431	0.252	0.000

C—conventional diet (C); E_1_–C+1% yeast; E_2_–C+2% parsley; E_3_–C+1% yeast and 2% parsley; data are presented as mean ± SEM; means within a column sharing different superscript are significantly different at: p < 0.05 or p < 0.001; hematocrit (HCT), leukocyte (LEUK), heterophyle (HET), lymphocyte (LYM), monocyte (MON), and eosinophil (EOS).

## 4. Discussion

### 4.1. Total polyphenol content and antioxidant capacity of experimented ingredients

As expected, higher values of total polyphenol content and antioxidant capacity were noticed on parsley compared to zinc-enriched yeast. Some authors (38) in a study on parsley juice obtained value of 14.87±1.03 (mg GAE/100 mL). As Lipiński et al. (39) stated polyphenols have antioxidant, antimicrobial, immunomodulatory, and anti-inflammatory functions, however, they present a low/poor bioavailability and absorption in poultry gut. Similar to our results, other authors (40) reported a parsley leaves total polyphenol concentration of 11.90 mg GAE/g for raw parsley leaves and 7.66 mg GAE/g, respectively. Different concentrations of lutein, zeaxanthin, and beta-carotene were obtained for parsley leaves containing 31.28 mg/100 g (41), 11.1 mg/100 g (42), and 32.83 mg/100 g (43), respectively. In a clinical study, it was stated that among the 50 foods with the highest antioxidant content, dried parsley (7.430 mmol/100 g) ranks the 14th (44).

### 4.2. Effects of heat stress on productive performances

Our study's results showed that dietary parsley and Zn-enriched yeast on E_3_ group registered a significant improvement of ADFI, ADEP, and ADW which conducted to a favorable FCR. Interesting results showed that after an initial decreasing of ADFI during the 2nd experimental week, the production parameter increased during the 3rd week by 5.7%, this indicating probably that hens started to accommodate with high temperatures. Other authors (45, 46) obtained similar results on poultry weight, feed intake, and egg production when adding dietary zinc in heat stress conditions. An enhanced egg yield and hatchability in quail when adding parsley leaf in poultry diets and a synergetic effect on feed consumption were observed by Tahan and Bayram, (47). Laying hens exposed to high, severe heat stress (32°C) for 42 days registered a significantly decreased ADFI by 18–22% compared to normal temperature group (22°C) or moderate temperature group (27°C) (48). Lara and Rostagno (14) stated that there is a large variability of heat stress effects on poultry experiments due to many factors involved (heat stress variation, temperature intensity, experimental length, humidity, poultry age, and genetics). Other authors (49) noticed that the decreasing percentage of laying hens' egg production (13.2, 26.4, and 57%, respectively) depends on the exposure time to heat stress (8–14 days, 30–42 days, and 43–56 days, respectively). Similar results (50) were obtained in a stress heat experiment on 24 weeks of age Hy-Line Brown hens where ADFI was 58.12 g/hen/day, average egg mass was 50 g, and FCR rate was recorded 1.29. Mashaly et al. (51) observed a 52% decreasing of ADFI when a constant heat stress was applied for 5 experimental weeks.

Ibtisham et al. (6) introduced a Chinese herbal medicine mixt (3.32 g/kg diet) and ginger powder (10 g/kg diet) and their combination in an experimental hall at 32–38°C in 3-tier cages layer houses (2 hens per cage) and noticed that production parameters as feed intake and egg production significantly improved compared to control group raised in heat stress conditions as well. Manaig et al. (52) state that plant extract utilization can provide a safe, accessible, and low-cost nutritional solution to heat stress problem by combatting the deleterious effects of heat stress on production performance. Büyükkiliç et al. (53) evaluated the effects of thyme essential oil (300 mg/kg) and vitamins A (15,000 IU/kg diet), C (250 mg/kg diet), and E (250 mg/kg diet) on laying hens' performance, egg quality, and biochemical parameters under 34°C heat stress (HS) for 8 h daily and noticed that the treatment had no effects on body weight, feed intake, egg production, feed conversion rate, egg weight, egg yield, and egg weight.

### 4.4. Internal and external egg parameter assessments during storage period

Within this study, we noticed that the whole egg weight was statistically significant higher (*p* < 0.05) on 0 day of storage period. Gradually, with the prolonged exposure on temperature ambience, the egg weight decreased due to the storage period losses. Same situation encountered for albumen, which had a significant higher (*p* < 0.05) weight for C, E_1_, E_2_ compared to E_3_ and for the first period of storage. Usually, an egg weight and egg shell thickness reduction in laying hens subjected to heat stress it can be noticed (3). Other studies (54) agree that egg weight decreased significantly with storage time and temperature. A significant loss of weight of eggs during storage may be a result of the increase of shells? spores while the egg aged which facilitate the moisture and gases to escape. The carbon dioxide escapes through the eggshells? pores which causes a white that becomes watery. In our study, egg yolk weight was statistically significant higher (*p* < 0.05) on the 14th and 28th period of storage, similar with the results obtained by other authors (55) who noticed that the yolks were heavier through 2 weeks of heat stress but becoming lighter analyzed at 4-week storage period. Fennel dietary inclusion (0, 10, or and 20 g/kg of diet) was tested under 34°C, and a significant decreasing egg quality, egg weight, eggshell thickness, eggshell strength, Haugh units, albumen height, and albumen weight were noticed by Gharaghani et al. (56). In addition, other authors (57) reported significant decreasing values of pH under heat stress conditions. The HU parameter decreased gradually with the lowest value on the 28th day. In another heat stress study (34°C for 20 days), a HU score reduction was reported by −5.6% compared to control group (58). Egg loss parameter is regarded as an important index of egg quality. Most studies carried out on egg storage conditions considered that egg weight loss gradually increased during storage and laying hens' age; therefore, egg quality deterioration is caused by CO_2_ and water losses in albumen through eggshells' pores (59). Concerning egg mass loss, similar results were obtained by other authors (60), who found that after 16 days of storage period a 2.65 % egg weight loss was registered, after 26-day storage period a 3.93 % egg weight loss was registered, and again after 31-day storage period a 4.72 % egg weight loss was noticed. According to the previous results, Jones et al. (61) stored unwashed eggs at room temperature and experienced 4.5 to 5.5% weight loss each month.

### 4.5. Effects of heat stress exposure on yolk color evolution

The intention of parsley dietary inclusion was mainly to mitigate heat stress negative effects on laying hens but using this ingredient came in conjunction with a more intense yolk coloring on E2 and E3 groups. It is well known that egg yolk is an important consumer criteria and using coloring natural sources, inexpensive and easy available with high antioxidants properties could be the optimum solution for obtaining a more intense color (59). Therefore, in our study, egg yolk color intensification was achieved by dietary inclusion of parsley. The main egg yolk color parameters evaluated were lightness (L^*^), redness (a^*^), and yellowness (b^*^). It was difficult to find similar studies on parsley to compare the results of our study with previous studies. However, there are authors (60) that found that a 1.5% parsley supplementation into laying quail diets resulted on a high coloration score of 11.3 on Hoffman La Roche scale.

### 4.6. Effects of dietary ingredients on TBARS values from egg yolk

Our results demonstrated that egg yolk TBARS values were statistically significant reduced (*p* < 0.05) during the 2nd and the 3rd experimental week by the addition of Zn-enriched yeast, parsley, and the mixture of the two ingredients. The antioxidant effects of parsley and Zn-enriched yeast influenced significant (*p* < 0.05) the TBARS values compared to C group, reducing the lipid degradation process of eggs stored at room temperature after being initially exposed to heat stress. Several studies conducted on laying hens and their response to different levels of heat stress by adding dietary ingredients to alleviate it (50, 61) demonstrated that high levels of oxidative products inside the birds' body will increase simultaneously in eggs composition. Thring et al. (62) suggested that phytogenic plant mode of action is capable of directly scavenging the stress-related ROS production, the pro-oxidant enzymes including lipoxygenase and NADPH oxidase.

### 4.7. Effects of heat stress on serum biochemical and hematological profiles

In our study, heat stress exposure had little effect on serum biochemical and hematological parameters except for Ca, Fe, GGT, HCT, and HET parameters. These results could be a physiologic evidence that biochemical and hematological parameters could normalize under prolonged heat exposure as animal thermoregulatory response (63). Other authors (64) observed an improvement on serum biochemical traits in broiler chicken when added parsley leafs. Usually, the increased number of white blood cells is seen as a response to bacterial infection. The increased EOS concentration registered on E3 group and also, HET increased concentration on C and E1 groups could be caused by the handling excitement. Thrall et al. (65) stated that blood collection process usually results in a physiologic leukocytosis which increases the concentration of HET and LYM in the peripheral blood, so not necessarily an inflammation process. Studies (66) found that exposure to a continuous heat stress (34.5 for 14 days of 22-day-old broiler) determined significant damage to LYM proliferation ion and differentiation which can cause immune abnormalities. In our experimental results, the UA registered a higher value on E1 group but without statistical significance (*p* > 0.05). Some authors (67) found that an increasing UA level can ameliorate free radicals and suppress lipid peroxidation. Contrary to our results, Omran (68) observed that HCT values decreased in heat stressed animals most probably due to erythrocytes destruction or hemodilution. Some authors (69, 70) noticed on 6-week-old male broilers that acute heat stress increases the Ca^2+^ in lymphocytes, knowing that calcium ions play an essential function in lymphocytes' activation and maturation. In general, the concentration of serum minerals (Mg, Ca, and P) is low, during heat stress, especially when the diets also are poor in these minerals, impairing the absorption and digestion of nutrients (48). Richmond and Mackley (71) stated that parsley has the ability to improve the cell immunity; therefore, the health status is enhanced.

## 5. Conclusion

Study limitation was represented by the experimental time period (4 weeks) in which the poultry had to resist at a permanent hall temperature settled at 34±1°C, during the entire experimental trial (24/24 h). We used 32 laying hens/group taking into consideration to avoid using a larger number of poultry respecting the 3Rs the main rule (replacement, reduction, and refinement), as stated in the EU Directive 2010/63/UE, which allows us to minimize animal distress and maintain animal welfare but being aware that the temperature is an essential/vital factor poultry welfare.

The results showed that the positive effects of Zn-enriched yeast and parsley, as single dietary inclusion or their combination, sustained egg production, egg mass and egg weight parameter performances, and egg quality during the heat stress study. The two additives demonstrated their antioxidant capacity by delaying the lipid peroxidation during different storage time periods. Taking into consideration the findings obtained from this heat stress study, we can design a nutritional strategy based on inclusion levels experimented within this study, in heat stress conditions, using affordable and efficient solutions to alleviate heat stress in laying hens. Further studies on laying hens are requested to experiment same ingredients, higher inclusion levels during heat stress conditions.

## Data availability statement

The raw data supporting the conclusions of this article will be made available by the authors, without undue reservation.

## Ethics statement

The experiment was conducted according to Directive 2010/63/EU, Executive Order No. 28/31.08.2011, Romanian Law No. 43/11.04.2014. The Experimental Protocol No. 118/02.12.2019 was approved by the Ethics Committee on Animal Experiments from the National Institute for Research and Development of Animal Biology and Nutrition, Balotesti, Romania.

## Author contributions

GC, TP, and AU contributed to the conceptualization. Methodology was assured by AU, IV, MD, MS, and PV. Software and data validation were realized by GC, TP, and AU. MD, MS, AU, and IV were involved in formal analysis. All authors contributed to manuscript revision, read, and approved the submitted version.

## Footnotes

^1^ https://eur-lex.europa.eu/legal-content/EN/TXT/PDF/?uri=CELEX:32019R0006
